# Schwannoma and Neurofibroma, Originating from the Ulnar Nerve in Neurofibromatosis: A Case Report and Review of the Literature

**DOI:** 10.1055/s-0040-1712536

**Published:** 2020-09-10

**Authors:** Ali Tabrizi, Ahmadreza Afshar, Iraj Mohebbi, Masoumeh Pourjabali, Hassan Taleb

**Affiliations:** 1 Department of Orthopedics, Imam Khomeini Hospital, Urmia University of Medical Sciences, Urmia, Iran; 2 Department of Occupational Health, Clinical Research Development Unit of Imam Khomeini Hospital, Urmia University of Medical Sciences, Urmia, Iran; 3 Department of Pathology, Imam Khomeini Hospital, Urmia University of Medical Sciences, Urmia, Iran

**Keywords:** schwannoma, neurofibroma, neurofibromatosis

## Abstract

Schwannomas and neurofibromas are rare benign tumors originating from the peripheral nerve sheath. Tumors in neurofibromatosis are mostly neurofibromas and often appear in the soft tissue of peripheral nerves. In this report, a patient presented with two large adjacent soft tissue tumors in the right wrist and distal forearm which originated from a common nerve. A schwannoma had formed beside a neurofibroma from the ulnar nerve and induced numbness and paresthesia in the little and ring fingers. Although the patient had café au lait spots on the skin, neurofibromatosis was not suspected due to lack of symptoms. The patient was referred to the current research clinic suffering from two soft tissue masses in the wrist and ulnar nerve dysfunction. In neurofibromatosis patients, two tumors of a different nature originating from a common nerve close together have rarely been described in the literature. The patient was treated by en bloc excision of the mass while protecting the nerve fascicles. The follow-up results indicated no neurological symptoms and complete restoration of ulnar nerve function.

The emergence of peripheral nerve sheath tumors (PNSTs) in the extremities is rarely reported. Benign PNSTs can be classified into two types: neurilemmoma (schwannoma) and neurofibroma (NF). 1 NFs are mainly seen in young patients aged between 20 and 40. In some cases, neurofibromatosis is more common in male children and adolescents. 1 2

NFs are one of the benign soft tissue tumors, accounting for at least 5% of soft tissue tumors. There are three types: localized, diffuse, and plexiform which is usually seen in neurofibromatosis. 2 A schwannoma is a benign neoplasm that originates from Schwann cells and is usually solitary; however, these soft tissue tumors can occur in large numbers in neurofibromatosis or schwannomatosis. 3 A wide range of peripheral nerve tumors can occur in neurofibromatosis patients. With solitary tumors and symptomatic patients, surgical excision is the definitive treatment and, in most cases, they are not likely to recur. 3 Surgical intervention is, however, controversial in cases with multiple tumors. We describe a patient presenting with a soft tissue mass in the wrist and forearm with ulnar nerve symptoms, numbness, and distal paresthesia. Two types of tumor were found, originating from the peripheral nerve.

## Case Report

**Fig. 1:**
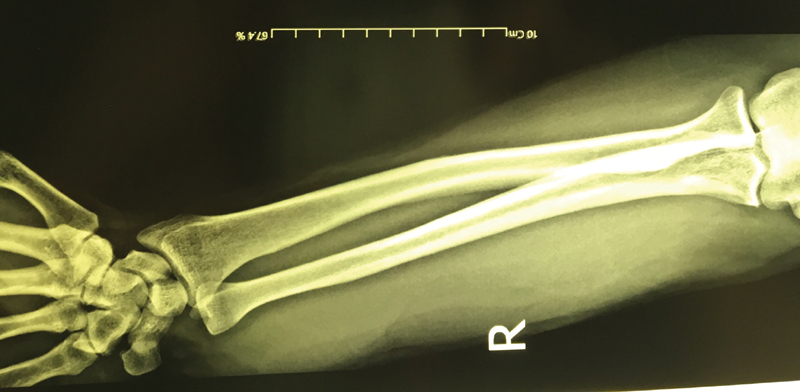
Conventional radiography of the right wrist and forearm showing a soft tissue mass and swelling in distal one-third of the forearm without bone involvement.

**Fig. 2:**
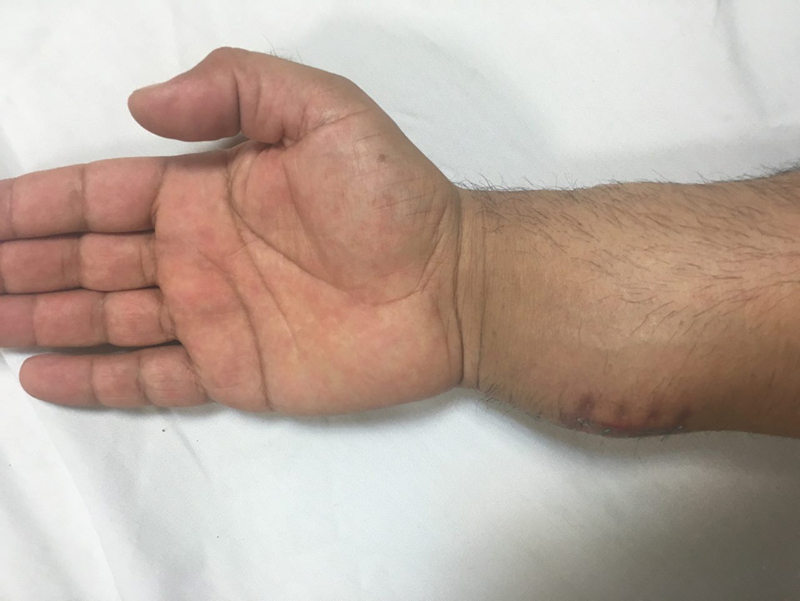
Clinical photo of the volar surface of the patient's right wrist and forearm with palpable firm masses.

A 25-year-old male was referred to our orthopedic clinic complaining of a palpable mass on the volar surface of his right wrist in the distal forearm. Examination showed a hard, palpable mass with an approximate dimension of 4 × 6 cm, attached to a second mass measuring 3 × 2 cm ( Figs. 1 and 2 ). The patient had no history of previous disease and had not been diagnosed before as he had no clinical symptoms. Circulation in the distal radial and ulnar arteries was normal. Neurological examination revealed paresthesia and numbness in the little and ring fingers; however, adduction and abduction were normal. Tinel's sign was positive in the ulnar nerve. Skin examination showed café au lait spots on the patient's back. There was no skeletal deformity.

**Fig. 3:**
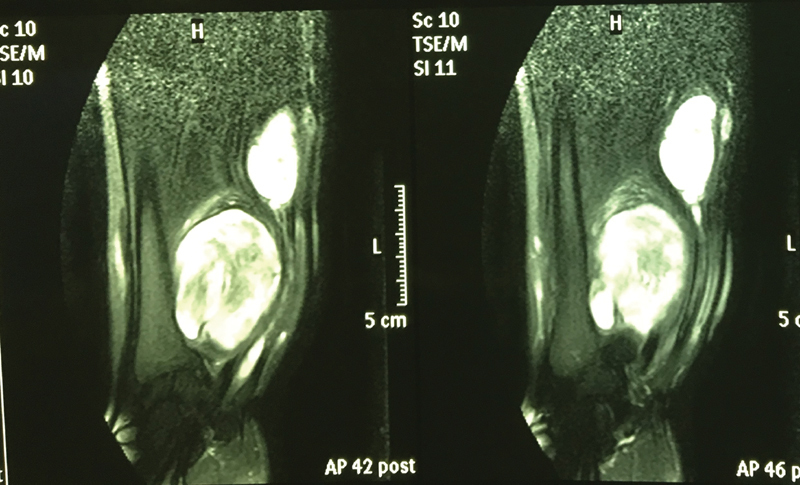
Magnetic resonance imaging (MRI) examination indicated two soft tissue tumors located by the ulnar nerve.

Magnetic resonance imaging (MRI) (Siemens Essenza 1.5T) was performed. A mass with a dimension of 6 × 5 cm ^2^ could be observed in T1-low and T2-high signals with poor enhancement; the second mass, 5.5 × 2 cm ^2^ , was located beside the former in the path of the ulnar nerve ( Fig. 3 ). An incisional biopsy was conducted through a 1-cm skin incision on the volar surface of the larger mass next to the ulnar styloid process. Histopathological investigation showed an NF. Because of the neurological symptoms, the dimensions of the tumor, and its pressure on the ulnar nerve, the patient was considered for the surgical removal of the mass. Based on the initial biopsy results, it was assumed that both masses seen on MRI were NFs.

**Fig. 4:**
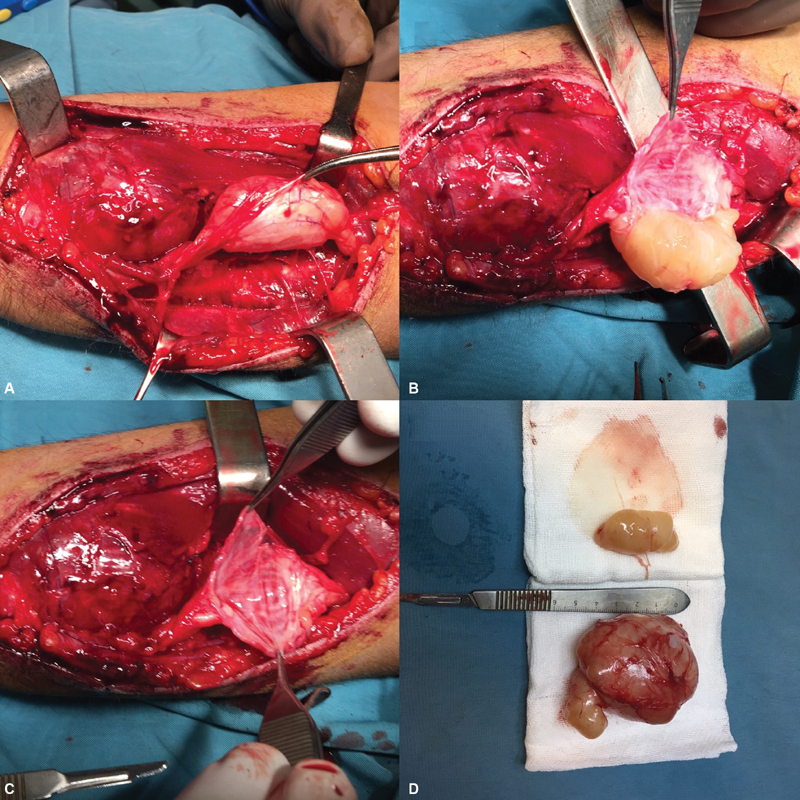
Intraoperative view showed: ( **A** ) tumors along the course of the ulnar nerve; ( **B** ) nerve sheath dissection; ( **C** ) operative field after removal of one of the tumors; ( **D** ) cross-section of the tumors.

**Fig. 5:**
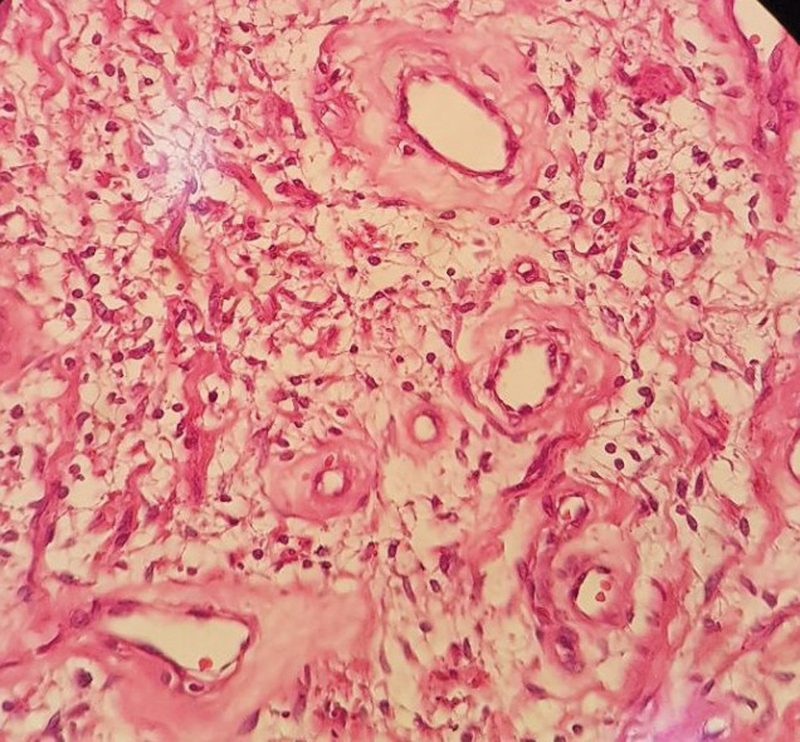
Histopathological examination results: H&E (Hematoxylin and Eosin) staining ×100 magnification; hypo and hypercellular overview (Antoni A&B) with vessel hyalinization.

**Fig. 6:**
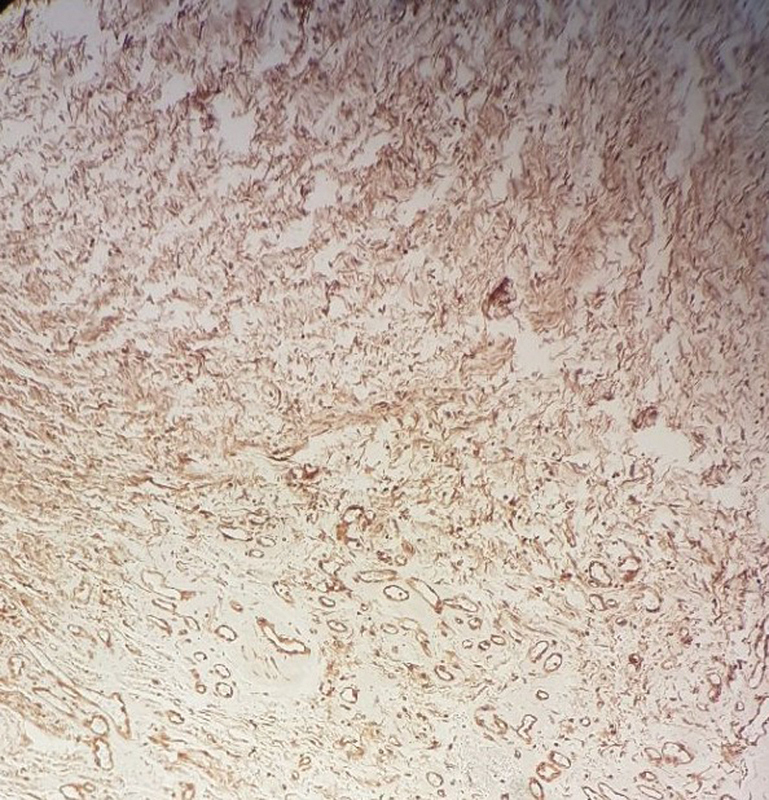
Immunohistochemical expression of S-100 protein in the Schwann cells.

**Fig. 7:**
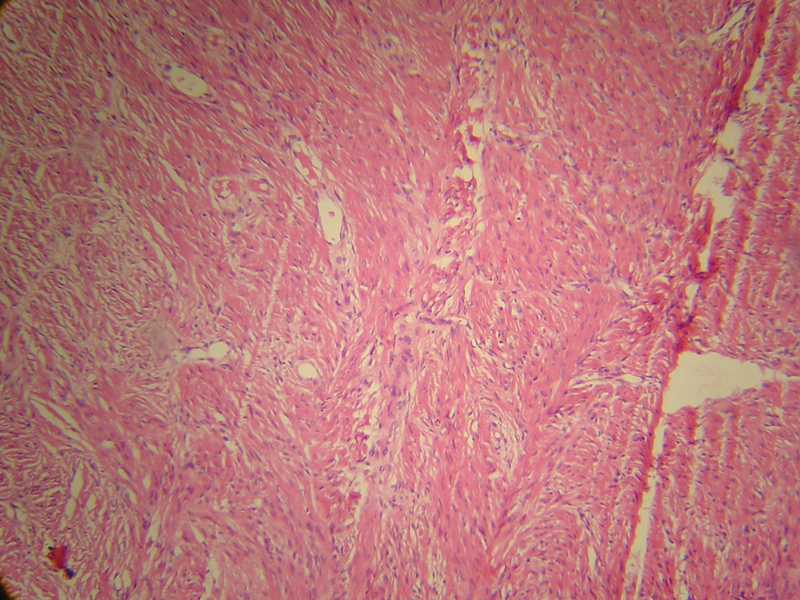
Microscopy (H&E Stain, ×100), indicating a spindle cell tumor with a fibromyxoid background of neurofibroma.

Under general anesthesia and with tourniquet-induced ischemia, the patient underwent microscope-assisted exploratory surgery. Through the volar approach on the ulnar side of the forearm, exploration was performed in the proximal to distal direction. A hard-fusiform mass was found in the ulnar nerve sheath in the distal one-third of the forearm on the volar surface, 7 cm proximal to the ulnar styloid process. It was closely attached to the larger mass found next to the ulnar styloid process, beside the ulnar nerve and intermuscular tendon expansion to the flexor digitorum superficialis muscles. The ulnar nerve sheath was carefully dissected from the nerve and the fusiform tumor was released from the nerve sheath without any damage to the fascicular structure of the nerve. The larger distal mass was completely removed by releasing it from the muscles and flexor tendons beside the ulnar nerve. Histopathological investigation with H&E staining ( Fig. 4 ) indicated a neurilemmoma, with an Antoni A structure, in the ulnar sheath. Microscopy showed polygonal cells with some regions containing spindle cells. According to immunohistochemical analysis, S-100 protein expression in the form of diffuse Schwann cells, which is indicative of schwannoma, was positive ( Figs. 5 and 6 ). Histopathological analysis of the larger mass showed a benign spindle cell tumor in the distal section, which suggested an NF ( Fig. 7 ). The immunohistochemical investigation of the tumor cells was negative for Desmin, SMA, BLC2, CD34, CD68, and CD31. Vimentin and S-100 protein were reported to be focally positive. B-catenin staining results revealed a cytoplasmic, but not nuclear, dot-like staining pattern. After the surgery and during the follow-up period, the patient's symptoms resolved completely and ulnar nerve function returned to normal despite the extensive dissection necessary during the surgery.

## Discussion

This study reports a patient with two soft tissue masses in his right wrist and distal forearm with ulnar nerve dysfunction who was treated by en bloc excision. These two different tumors originated from a common nerve, which makes the current case report unique. The patient had café au lait spots on his back and despite having had no problems until adolescence, the investigations showed that he met the criteria for neurofibromatosis type-1 (NF-1). There are two types of NF, type-1 is characterized by neurocutaneous syndromes 4 5 and PNSTs are found in as many as 30% of the cases. 6 Benign tumors include schwannomas, NFs, and the pathognomonic plexiform NFs. 7

The tumors of the peripheral nerve sheath in NF-1 are mostly benign NFs 4 ; however, malignant transformation can occur in up to 38% and they are deeply located and usually clinically silent tumors. 8 Malignant peripheral nerve sheath tumors usually occur in NF-1, 4 8 therefore, these patients should be fully informed about their propensity to develop malignant PNSTs and should be urged to have regular check-ups and seek medical advice whenever symptoms related to malignancy manifest. Unfortunately, many patients with NF-1 are undiagnosed or inappropriately treated, especially when health care is not easily accessible. Nerve tumors are frequently mismanaged; the fear of neurological complications may result in them being conservatively managed or removal of symptomatic tumors may be performed by surgeons without expertise in the field. The prevention of malignancy and efforts to ensure its early detection are often neglected even though NF-1 patients are known to present a lifetime risk of developing malignant PNSTs that ranges from 8 to 13%. 9

NFs can be superficial or deep and can grow beside any peripheral nerve. 4 Their natural history varies greatly, and they can remain symptomless or induce progressive pain, weakness, tingling, and numbness. Soft tissue tumors are often the first clinical findings. 10 A schwannoma is a benign tumor originating from the Schwann cells of the nerve sheath. 3 Schwannomas in the hand and wrist are uncommon, accounting for approximately 7.5% of schwannoma cases. 3 The incidence of schwannoma in NF-1 is unknown, although it is rare in NF. 11 Schwannomas have been reported in the superficial radial nerve in several studies.

The first case of a schwannoma in the superficial radial nerve was reported by Visser. It was in the wrist, located 7 cm from the styloid process of the radius. 12 An ulnar nerve schwannoma has also been reported by Van Herendael et al. 13 In most cases, wrist and forearm schwannomas arise in the median nerve and are rarely seen in the ulnar nerve. In a report by Cervoni et al, a fusiform mass in the ulnar nerve of a 44-year-old man was treated by wide en bloc excision, and complete functional improvement of the ulnar nerve was observed after 13 months of follow-up. 14 The excision of a schwannoma arising from a major peripheral nerve can be conducted with an acceptable risk of nerve injury, although a transient neurological deficit is often expected. 15 This is the first report of two different types of tumors arising beside each other. A primary biopsy of the larger mass led to the diagnosis of NF, whereas the histological diagnosis of the different second tumor was made after the surgery. Surgical excision led to a favorable outcome for the patient.

A study by Gosk et al in 2015 addressed schwannoma surgery in limb extremities. 16 Examination of 35 patients who had undergone peripheral nerve schwannoma surgery and tumor removal showed that they all recovered fully after excision. The probability of a neurological deficit is very low. 16 This combination of tumors is called a hybrid tumor. Murărescu et al reported an unusual peripheral nerve sheath that had histological and immunohistochemical features of a NF and schwannoma in a single mass. 17 In a report by Hussain et al in 2016, a hybrid tumor was found in an NF-1 patient who was treated with surgical excision. 18 Similar conditions that triggered the hybrid tumor appear to have existed in our patient; however, in this case, the adjacent tumors originated from a common nerve sheath.

In this patient, the neurological symptoms were completely resolved by surgical excision with nerve protection and the ulnar nerve function was fully restored. This was achieved by careful dissection of the nerve sheath to protect the nerve fascicle. Surgical excision with complete tumor removal is the best treatment for tumors originating from the peripheral nerve. About 10 to 23% of solitary fibrous soft tissue tumors could have malignant behavior. 19 They are unpredictable and need long-term follow-up after surgical tumor removal. 20 However, in the current case, two separate but adjacent nerve sheath tumors originated from the ulnar nerve. One of the predisposing factors for such a condition is neurofibromatosis, which can produce a wide range of PNSTs highlighting the need for careful preoperative examination.

## Conclusion

In patients with neurofibromatosis, different types of soft tissue tumors can originate from the peripheral nerves. This is the first report of a NF and schwannoma growing beside each other originating from the same nerve (ulnar nerve). Unfortunately, many patients with NF-1 are still undiagnosed or inappropriately treated, especially where health care is not easily accessible.
